# Electronic Cigarettes as an Introductory Tobacco Product Among Eighth and 11th Grade Tobacco Users — Oregon, 2015

**DOI:** 10.15585/mmwr.mm6623a2

**Published:** 2017-06-16

**Authors:** Jonas Z. Hines, Steven C. Fiala, Katrina Hedberg

**Affiliations:** ^1^Epidemic Intelligence Service, CDC; ^2^Public Health Division, Oregon Health Authority.

During 2011–2015, increased electronic cigarette (e-cigarette) and hookah use offset declines in cigarette and other tobacco product use among youths (persons aged <18 years) (*1*). Limited information exists about which tobacco product introduced youths to tobacco product use. Patterns of first use of e-cigarettes among Oregon youths who were tobacco users were assessed in the Oregon Healthy Teens 2015 survey, a cross-sectional survey of eighth and 11th grade students in Oregon. Respondents were asked, “The very first time you used any tobacco or vaping product, which type of product did you use?” Among students who had ever used any tobacco product (ever users), e-cigarettes were the most common introductory tobacco product reported by both eighth (43.5%) and 11th (34.4%) grade students. Among students who used a tobacco product for ≥1 day during the past 30 days (current users), e-cigarettes were the most common introductory tobacco product reported by eighth grade students (44.4%) and the second most common introductory tobacco product reported by 11th grade students (31.0%). Introductory use of e-cigarettes was commonly reported among youths in Oregon who were ever or current tobacco users, underscoring the importance of proven interventions to prevent all forms of tobacco use among youths (*2*,*3*).

Tobacco use is the leading cause of preventable disease and death in the United States, and the majority of adult cigarette smokers first try smoking before age 18 years (*2*). During the past 3 decades, cigarette smoking among youths has declined substantially, in both Oregon and nationally (*3*–*5*). However, during 2011–2015, increased electronic cigarette and hookah use offset declines in cigarette and other tobacco product use among youths nationally; in 2014, e-cigarettes surpassed cigarettes as the most commonly used tobacco product among youths (*1*).

Among youths, use of e-cigarettes is strongly associated with use of other tobacco products, including combustible tobacco products (*3*,*6*). In 2015, the majority of students in U.S. middle and high school who used combustible tobacco (including conventional cigarettes) concurrently used e-cigarettes; however, which type of tobacco product these students are likely to use first remains unknown (*3*). Limited information exists about which product was used as an introduction to tobacco products after e-cigarettes became commonly used among U.S. youths (*7*). Using data from the Oregon Health Teens surveys, patterns of first use of e-cigarettes were assessed among youths in Oregon who were tobacco users.

Oregon Healthy Teens is a cross-sectional, school-based, biennial survey of health behaviors administered to Oregon eighth and 11th grade students. A statewide representative sample is obtained from a random sample of public high schools and their feeder middle schools, stratified by county. Students’ parents are notified before survey administration and can decline participation for their child. Students can opt out of participating at the time of survey administration. Responses are anonymous, and data are weighted based on statewide school enrollment numbers to represent students across Oregon proportionally. During February–May 2015, a total of 16,104 eighth grade and 13,570 11th grade students participated in the surveys; response rate was 83% among 308 schools that were contacted for survey recruitment.

In 2015, respondents were asked, “The very first time you used any tobacco or vaping product, which type of product did you use?” Response options included the following: I have never used any tobacco or vaping product; cigarette; chewing tobacco; small cigar; large cigar; hookah; e-cigarette or other vaping product; and another type of product. The introductory tobacco product used was assessed among ever and current tobacco product users. Respondents were considered ever users if they indicated tobacco product use for the following survey questions: “How old were you when you smoked a whole cigarette for the first time?” or “How old were you when you first used any form of tobacco other than cigarettes?” Respondents were considered current users if they indicated use of a tobacco product ≥1 day during the past 30 days. Tobacco products were categorized as cigarettes, e-cigarettes, hookahs, and other tobacco products (small cigars, large cigars, chewing tobacco, and unspecified tobacco products).

In 2015, among Oregon eighth grade students, 21.9% reported having ever used any tobacco product and 12.3% reported current use; among Oregon 11th grade students, 41.7% reported having ever used any tobacco product, and 23.7% reported current use. E-cigarettes were the most common introductory tobacco product among ever (43.5%) and current (44.4%) eighth grade users (Table). Among 11th grade users of any tobacco product, e-cigarettes were the most commonly reported introductory tobacco product among ever users (34.4%) and the second most commonly reported introductory product among current users (31.0% of current users reported first using e-cigarettes and 31.1% reported first using conventional cigarettes).

**TABLE:** Introductory tobacco products used among eighth and 11th grade students who ever used or currently use any tobacco product and cigarettes — Oregon Healthy Teens Survey, 2015

School grade	Introductory product	Ever user % (95% CI)		Current user % (95% CI)		
		Any tobacco	Cigarettes	Any tobacco	Cigarettes	Cigarettes and e-cigarettes
8	E-cigarettes	43.5 (39.9–47.2)	25.1 (21.9–28.5)	44.4 (40.8–48.2)	22.2 (18.3–26.7)	30.5 (25.4–36.1)
	Cigarettes	27.2 (23.7–30.9)	48.7 (43.5–53.9)	25.0 (21.4–29.0)	53.9 (47.5–60.3)	44.1 (37.0–51.4)
	Hookah	16.7 (13.6–20.2)	11.9 (9.5–14.8)	16.9 (12.9–21.9)	9.9 (7.0–13.7)	12.2 (8.9–16.4)
	Other tobacco product*	12.6 (10.5–15.1)	14.3 (10.5–19.3)	13.6 (11.3–16.3)	14.0 (9.7–19.8)	13.3 (8.6–20.0)
11	E-cigarettes	34.4 (31.9–37.0)	17.7 (15.4–20.4)	31.0 (28.2–34.0)	14.7 (10.6–19.9)	15.4 (10.7–21.7)
	Cigarettes	29.6 (27.3–32.0)	52.6 (49.5–55.7)	31.1 (28.5–33.7)	57.9 (52.4–63.1)	57.1 (50.3–63.7)
	Hookah	18.8 (17.1–20.5)	12.8 (11.1–14.8)	15.8 (13.7–18.1)	10.4 (8.4–12.7)	10.0 (7.8–12.7)
	Other tobacco product*	17.2 (15.4–19.2)	16.8 (14.8–19.1)	22.1 (19.5–25.0)	17.1 (14.2–20.5)^†^	17.5 (14.2–21.3)^†^

**Abbreviation:** CI = confidence interval.

* Other tobacco products include cigars, large cigars, chewing tobacco, or unspecified.

^†^ Percent reflects total for composite variable (i.e., other tobacco product); however, when examined by individual introductory product, e-cigarettes were the second most common introductory tobacco product among 11th grade students who were current cigarette users, regardless of concurrent e-cigarette use.

Among eighth and 11th grade students who were conventional cigarette users, e-cigarettes were the second most common introductory tobacco product among ever (25.1% and 17.7%, respectively) and current (22.2% and 14.7%) users (Table). Among current conventional cigarette users who currently also used e-cigarettes, e-cigarettes were the second most common introductory tobacco product for both eighth (30.5%) and 11th grade students (15.4%).

## Discussion

In 2015, e-cigarettes were the most common introductory tobacco product used among Oregon eighth and 11th grade students who had ever tried tobacco products. E-cigarettes were also a common introductory tobacco product for current conventional cigarette users among eighth and 11th grade students in Oregon. Although e-cigarettes were a commonly reported introductory product in both grades, the lower prevalence of introductory use of e-cigarettes among 11th grade students might reflect tobacco use initiation that occurred before the widespread availability of e-cigarettes. This study extends reports on the increases in e-cigarette use by examining introductory tobacco products among youths who were users of tobacco products. However, further studies are needed to establish temporality of e-cigarette and conventional tobacco product use among youths.

The findings in this report are subject to at least four limitations. First, the data were self-reported, and therefore, subject to recall and reporting bias. Second, observational data do not allow for evaluation of a causal link between e-cigarette use and initiation of cigarette smoking. Third, because the survey question of interest was first asked in 2015, it is not possible at this time to report a trend in introductory tobacco products. Finally, data are only collected from eighth and 11th grade students who attend public schools and are therefore not representative of all Oregon youths.

Introductory use of e-cigarettes was commonly reported among youths in Oregon who were ever or current tobacco users. A 2016 Surgeon General’s report concerning e-cigarettes concludes that use of nicotine-containing products in any form, including e-cigarettes, among youths is unsafe (*3*). The report notes that action can be taken at the national, state, local, tribal, and territorial levels to address e-cigarette use among youths and young adults. Public health interventions could include smoke-free policies that include e-cigarettes, restrictions on youths’ access to e-cigarettes, pricing strategies, retail licensure, regulation of e-cigarette marketing likely to attract youths, and educational initiatives focused toward youths and young adults (*3*). CDC has issued evidence-based guidelines to establish comprehensive tobacco control programs, and in 2016, the Food and Drug Administration finalized rules extending its regulatory authority of tobacco products to include e-cigarettes (*8*,*9*). The findings of this study underscore the importance of proven interventions to prevent all forms of tobacco use, including e-cigarette use, among youths.

**Summary**

**What is already known about this topic?**

Electronic cigarette (e-cigarette) use among youths is strongly associated with use of other tobacco products, including combustible tobacco products. Limited information exists about which tobacco product introduced youths to the use of tobacco products after e-cigarettes became widely available in the late 2000s.

**What is added by this report?**

In 2015, e-cigarettes were commonly reported as the introductory tobacco product among youths who had ever used or currently use any tobacco product and cigarette smokers in eighth and 11th grades in Oregon.

**What are the implications for public health practice?**

The findings of this study underscore the importance of proven interventions to prevent all forms of tobacco use, including e-cigarettes, among youths.

## References

[1] Singh T, Arrazola RA, Corey CG, Tobacco use among middle and high school students—United States, 2011–2015. MMWR Morb Mortal Wkly Rep 2016;65:361–7. 10.15585/mmwr.mm6514a1 27077789

[2] US Department of Health and Human Services. Preventing tobacco use among youth and young adults: a report of the Surgeon General. Atlanta, GA: US Department of Health and Human Services, CDC; 2012. https://www.surgeongeneral.gov/library/reports/preventing-youth-tobacco-use/index.html

[3] US Department of Health and Human Services. E-cigarette use among youth and young adults. A report of the Surgeon General. Atlanta, GA: US Department of Health and Human Services, CDC; 2016. https://www.cdc.gov/tobacco/data_statistics/sgr/e-cigarettes/index.htm

[4] Oregon Public Health Division. Tobacco fact sheet, 2014. Portland, OR: Oregon Health Authority, Tobacco Prevention and Education; 2014. https://public.health.oregon.gov/PreventionWellness/TobaccoPrevention/Documents/countyfacts/OHA-Oregon-TobaccoFactSheet.pdf

[5] Kann L, McManus T, Harris WA, Youth risk behavior surveillance—United States, 2015. MMWR Surveill Summ 2016;65:1–174. 27280474 10.15585/mmwr.ss6506a1

[6] Miech R, Patrick ME, O’Malley PM, Johnston LD. E-cigarette use as a predictor of cigarette smoking: results from a 1-year follow-up of a national sample of 12th grade students. http://tobaccocontrol.bmj.com/content/early/2017/01/04/tobaccocontrol-2016-053291 10.1136/tobaccocontrol-2016-053291 PMC5545162 28167683

[7] Ambrose BK, Day HR, Rostron B, Flavored tobacco product use among US youth aged 12–17 years, 2013–2014. JAMA 2015;314:1871–3. 10.1001/jama.2015.13802 26502219 PMC6467270

[8] CDC. Best practices for comprehensive tobacco control programs—2014. Atlanta, GA: US Department of Health and Human Services, CDC; 2014. https://www.cdc.gov/tobacco/stateandcommunity/best_practices/pdfs/2014/comprehensive.pdf

[9] Food and Drug Administration. Deeming tobacco products to be subject to the Federal Food, Drug, and Cosmetic Act, as amended by the Family Smoking Prevention and Tobacco Control Act; restrictions on the sale and distribution of tobacco products and required warning statements for tobacco products. Rockville, MD: US Department of Health and Human Services, Food and Drug Administration; 2016. https://www.gpo.gov/fdsys/pkg/FR-2016-05-10/pdf/2016-10685.pdf

